# Protein expression profile and prevalence pattern of the molecular classes of breast cancer - a Saudi population based study

**DOI:** 10.1186/1471-2407-10-223

**Published:** 2010-05-21

**Authors:** Dalal M Al Tamimi, Mohamed A Shawarby, Ayesha Ahmed, Ammar K Hassan, Amal A AlOdaini

**Affiliations:** 1 Pathology Department, College of Medicine, University of Dammam, Dammam, Saudi Arabia; 2 Department of Biostatistics & Genetic Epidemiology, College of Medicine, University of Dammam, Saudi Arabia

## Abstract

**Background:**

Breast cancer is not a single entity but a diverse group of entities. Advances in gene expression profiling and immunohistochemistry as its surrogate marker have led to the unmasking of new breast cancer molecular subtypes, resulting in the emergence of more elaborate classification systems that are therapeutically and prognostically more predictive. Molecular class distribution across various ethnic groups may also reveal variations that can lead to different clinical outcomes in different populations.

**Methods:**

We aimed to analyze the spectrum of molecular subtypes present in the Saudi population. ER, PR, HER2, EGFR and CK5/6 were used as surrogate markers for gene expression profiling to classify 231 breast cancer specimens. Correlation of each molecular class with Ki-67 proliferation index, p53 mutation status, histologic type and grade of the tumor was also carried out.

**Results:**

Out of 231 cases 9 (3.9%) were classified as luminal A (strong ER +ve, PR +ve or -ve), 37 (16%) as luminal B (weak to moderate ER +ve, and/or PR +ve), 40 (17.3%) as HER2+ (strong or moderately positive HER 2 with confirmation by silver enhanced *in-situ *hybridization) and 23 (10%) as basal (CK5/6 or EGFR +ve). Co-positivity of different markers in varied patterns was seen in 23 (10%) of cases which were grouped into a hybrid category comprising luminal B-HER2, HER2-basal and luminal-basal hybrids. Ninety nine (42.8%) of the tumors were negative for all five immunohistochemical markers and were labelled as unclassified (penta negative). A high Ki-67 proliferation index was seen in basal (p = 0.007) followed by HER2+ class. Overexpression of *p53 *was predominantly seen in HER2 + (p = 0.001) followed by the basal group of tumors. A strong correlation was noted between invasive lobular carcinoma and hormone receptor expression with 8 out of 9 lobular carcinoma cases (88.9%) classifiable as luminal cancers. Otherwise, there was no association between the molecular class and the histologic type or grade of the tumor.

**Conclusions:**

Subtyping by use of this immunohistochemical panel revealed a prevalence pattern that is unique to our population; luminal tumors comprised only 19.9%, and the unclassified group (penta negative) 42.8%, a distribution which is distinctive to our population and in contrast with all Western studies. The presence of a predominant unclassified group also suggests that the currently used molecular analytic spectrum may not completely encompass all molecular classes and there is a need to further refine and develop the existing classification systems.

## Background

Breast cancer is heterogenous in its presentation, showing marked diversities spanning its morphological features, clinical outcomes, intrinsic subtypes and prevalence patterns. In Western countries, breast cancer is the most common female cancer and the leading cause of cancer mortality [1]. In the Kingdom of Saudi Arabia (KSA), although its incidence is lower than in Western countries [2], it is ranked highest amongst all the malignancies seen in Saudi females, comprising 21.8% [3]. Breast cancer in Saudi women displays features and characteristics that make it distinct from what is seen in Western nations [4]. Breast cancers in Saudi women are generally of a high grade, are locally advanced at the time of diagnosis, and affect predominantly females between 46-50 years of age, which is noticeably different from the median of 60-65 years seen in industrialized Western nations [5], where locally advanced disease is much less common.

The existing histological classification systems for breast cancer are far from being accurate in predicting the prognosis of a given patient [6]. Morphologically identical tumors can display divergent clinical outcomes. This can predominantly be attributed to molecular class differences that exist amongst the histologically similar types. A detailed knowledge about these molecular classes and their specific identification could lead to the development of a diagnostically more advanced and prognostically more beneficial reporting system that could offer much more precise prediction scores [7]. For this reason, there is an urgent need to learn more about the underlying biology of this disease [8].

The development of molecular analytical methods dates back to a quarter of a century, when immunohistochemistry (IHC) first allowed us to segregate these tumors into two main classes: ER+ and ER-. A decade later, the next step forward was the emergence of nucleic acid *in situ *hybridization. This led to the identification of two new categories, dependent on whether HER2 was amplified or not. Many other single gene molecular markers were assessed, but failed to prove clinically relevant [9]. Further progress in this area led to development of gene expression profiling, providing the simultaneous quantification of multiple genes; the final results could then be combined and used as a more reliable prognostic and predictive score [7].

Perou *et al. *were the first to draft a classification system based on gene expression analysis, and this consisted of four major molecular classes of breast cancer: luminal-like, basal-like, normal-like, and HER-2 positive [10]. Subsequent studies suggested the existence of more molecular classes [11-13] and this ultimately led to addition of a fifth subtype, with the molecular analytic spectrum now expanding to luminal A (LUMA), luminal B (LUMB), human epidermal growth factor receptor-2 (HER2) overexpressing, basal-like, and normal-like [12].

A further advancement in this field was the use of IHC as a surrogate marker for gene expression profiling. Studies confirmed that it could reliably identify the major molecular subgroups of invasive breast carcinoma [14-16]. Recently published studies have used five IHC surrogate markers (ER, PR, HER2, CK5/6, and EGFR) for molecular class distinction [17-19], with luminal tumors being categorized by hormone receptor (HR) positivity, HER2 expression a feature of HER 2 tumors, and CK5/6 and/or EGFR indicative of basal-like tumors. The molecular dimensions were further diversified by the introduction of a hybrid category comprising the HER2 + luminal tumors [20].

Diversities in the classification systems continue to emerge, but current studies still lack the potential to formulate a simple, practical, and easily applicable classification system for gene expression analysis that can completely and concisely encompass all the divergent facets of breast cancer [20]. In addition, the major studies conducted so far are predominantly reflective of breast cancer patterns, subtypes, and behaviour in Western populations; there remains the need to explore these issues in different regions.

We initially conducted a pilot study in which gene expression profiling by real time quantitative PCR was used to determine the "intrinsic" Saudi breast cancer subtypes and its prevalence was compared to the more commonly profiled Caucasian population [19]. A comparison between the IHC profiles of these breast cancers was correlated to the gene expression analysis and a discrepancy rate of 39% was identified, most conspicuously in the luminal type. That work suggested that breast cancer from Saudi and Caucasian populations may have a similar biology, but show variability in the subtype distributions. It was concluded that analysis of a larger cohort of patients was needed to precisely determine the molecular taxonomy of breast cancer in the Saudi population.

The present study used a larger regional based cohort, and the spectrum of molecular patterns seen in the Saudi population was analyzed by combining and modifying the current and upcoming molecular trends. The prevalence patterns obtained were compared and contrasted with Western patterns and other regionally based studies. A correlation of each subtype with Ki-67 proliferation index, p53 mutation status, histological type and grade of the tumor was also carried out.

## Methods

Breast carcinoma cases of Saudi patients were retrieved from the files of the Pathology Department of King Fahd Hospital of the University (KFHU) under the approved protocols of the research ethical committee of University of Dammam. Patient consent was waived as many of the cases were lost to follow up. Located in the Eastern Province of KSA, KFHU is one of the leading teaching hospitals in the Kingdom. Two hundred and thirty one cases were randomly selected for this study based on the availability of representative blocks and sufficient tissue material to perform the required procedures. The time frame covered was 12 years (1997 - 2008). Out of the 231 cases, 92 (39.8%) were mastectomy specimens, 53 (23%) were lumpectomies, and 86 (37.2%) were tru-cut biopsies. The age of the patients ranged from 25 to 97 years with a mean of 49.5 years (SD ± 11). The histologic type (according to WHO classification) and grade of the tumor (according to the modified Scarff-Bloom-Richardson grading system) were recorded as reported in the original pathology reports. Representative cancerous tissue obtained from paraffin blocks of mastectomy and lumpectomy cases were incorporated into 5 tissue microarray (TMA) reception blocks, from which sections were cut for IHC and *in situ *hybridization studies. For tru-cut biopsies, conventional paraffin blocks were utilized. IHC analysis for 5 markers (ER, PR, HER2, EGFR (HER1), and CK5/6) was used as a surrogate for gene expression profiling to classify the carcinomas into molecular classes. The prevalence of the different classes was then compared to that reported in large Western and other regional studies. Moreover, each class was correlated with its Ki-67 proliferation index and p53 gene over-expression, as revealed by IHC, and also with the histologic type and grade of the tumor.

### TMA construction

Two representative cancerous foci were marked on slides with H & E-stained sections from the selected paraffin blocks. TMAs were then created using two 0.6 mm tissue cores from each block that were punched out and inserted into the recipient TMA paraffin block using an Advanced Tissue Arrayer, Model VTA-100 (Veridiam, USA).

### Immunohistochemistry

Immunohistochemical staining using the labeled streptavidin-biotin (LSAB) method with 3,3'-diaminobenzidine (DAB) as a chromagen was performed for ER, PR, HER2, EGFR (HER1), CK5/6, Ki-67 and p53 on 4 μm thick paraffin sections cut from TMA and conventional blocks. The staining was performed concurrently in a Ventana Benchmark automated immunostainer according to the manufacturer's instructions (Ventana Medical Systems Inc., Strasbourg). Sources and dilutions of the primary antibodies used in the study are listed in Table 1. The immunostained sections were examined under a light microscope and evaluated manually by 2 pathologists (MS and DT). In case two TMA tissue cores were scored differently, conclusive scoring was made on a conventional paraffin section from the tumor. Any interpretational discrepancies were resolved under a double-headed microscope.

**Table 1:** Sources and dilutions of primary antibodies used in the study.

Antibody	Clone	Manufacturer	Dilution
ER	1D5	Dako	1:200

PR	PgR 636	Dako	1:50

HER2	CB11	Ventana	Prediluted

CK5/6	D5/16 B4	Dako	1:50

EGFR	H11	Dako	1:200

Ki67	MIB-1	Dako	Prediluted

P53	D0-7	Novacastra	Prediluted

ER = estrogen receptor, PR = progesterone receptor, HER2 = human epidermal growth factor receptor 2, CK5/6 = cytokeratin 5/6, EGFR = epidermal growth factor receptor, Ki67 = Ki67 nuclear antigen, P53 = human P53 protein

### Interpretation of immunohistochemical results

#### ER & PR

Results were reported using a semiquantitative score (H score) as described by Bhargava *et al. *[20], which assesses the percentage of positive nuclei (cytoplasmic staining considered negative) into categories of no staining, weak, moderate, or strong staining. The score was given as the sum of the percent staining multiplied by an ordinal value corresponding to the intensity level (0 = none, 1 = weak, 2 = moderate, 3 = strong). With four intensity levels, the resulting score ranged from 0 (no staining) to 300 (strong diffuse staining). A score of 10 or less was considered negative, a score of 11 - 99 weakly positive, a score of 100 - 199 moderately positive, and a score of 200 - 300 strongly positive.

#### HER 2/neu

Results were recorded according to the new guidelines set by the American Society of Clinical Oncologists/College of American Pathologists (ASCO/CAP) [21]. Positivity for HER2 was confirmed by silver enhanced *in situ *hybridization (SISH) for HER2 gene amplification as detailed below.

#### EGFR

Positive staining was defined as positive membrane staining, and was scored according to the criteria originally developed for HER 2/neu into negative, 1+, 2+ and 3+, using 10% staining of tumor cells as the cut off point [22].

#### CK5/6

Positive staining was defined as cytoplasmic staining with perinuclear enhancement. A staining intensity index was used, defined as the product of staining intensity (0-3) and proportion of immunoreactive cells (less than 10% = 1, 10-50% = 2, more than 50% = 3). Specimens with staining indices 1-9 were defined as positive, those with a staining index of 0 were defined as negative [23].

#### Ki-67

Positive staining was defined as positive nuclear staining. Cytoplasmic staining was considered negative. The percentage of positive nuclei was expressed as a "Ki-67 labeling index" which is the percent of cells expressing Ki-67 determined by counting 1000 cells/slide The percentage of positive cells was scored as follows: less than 10% = low proliferative activity, 10-40% = moderate proliferative activity, and more than 40% = high proliferative activity [24].

#### P53

Postive staining was defined as positive nuclear staining. Cytoplasmic staining was considered negative. Tumors were considered focally positive when unequivocal staining was present in 10-50% of tumor cells, and as diffusely positive when more than 50% of the tumor cells were positive [25].

### Silver enhanced *In Situ *Hybridization (SISH)

SISH for HER2 gene amplification was performed on paraffin sections prepared from TMA and conventional blocks in a Ventana Benchmark IHC/ISH instrument (Ventana Medical Systems Inc., Strasbourg) according to the manufacturer's instructions. The results were evaluated by light microscopy under a 40× objective. HER2 was considered positive if there was a gene amplification with a ratio of HER2 to the chromosome 17 centromeric region of more than 2.2. Equivocal SISH results (ratio 1.8-2.2) were considered negative for HER2.

### Tumor classification

**Figure 1:**
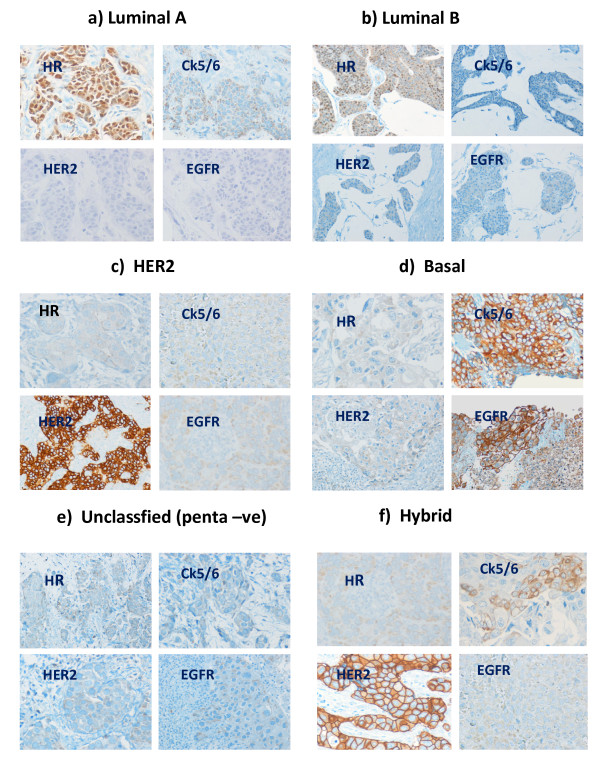
**Tumor classification (original magnification × 200)**. a) Luminal A: ER strongly positive, PR positive or negative, HER2 negative, CK5/6 and EGFR negative. b) Luminal B: ER weakly to moderately positive and/or PR positive, HER2 negative, CK5/6 and EGFR negative. c) HER2: HER2 positive, ER and PR negative, CK5/6 and EGFR negative. d) Basal: CK5/6 and/or EGFR positive, ER and PR negative, HER2 negative. e) Unclassified (penta-ve): ER and PR negative, HER2 negative, CK5/6 and EGFR negative. f) Hybrid: co-positivity of different markers in varied combination, an example of HER2-Basal hybrid (HBH) is shown.

The tumors were classified into five main categories as follows: LUMA, LUMB, HER2, basal-like, unclassified (UC/penta-ve), and hybrid. The hybrid type was further divided into four subtypes, Luminal B-HER2 (LBHH), Luminal A-Basal (LABH), Luminal B-Basal (LBBH) and HER2-Basal (HBH) Hybrids. The criteria for each class are shown in Figure 1.

Our criteria are generally similar to previous studies that used IHC as a surrogate for gene expression profiling [17,18,20], though primarily corresponding to those used by Bhargava *et al. *[20].

### Statistical Analysis

Data were entered into the computer using SPSS for windows. Comparisons between means and percentages were done via the student's t-test, ANOVA, chi-square and Fisher's exact test as appropriate. P < 0.05 was regarded as significant. Frequency tables, cross-tabulation, and measures of means, medians, standard deviations, and graphs were performed as descriptive statistics.

## Results

### Prevalence of molecular types

Of the 231 breast carcinoma cases examined, LUMA comprised 9 (3.9%), LUMB 37 (16.0%), HER2 40 (17.3%), basal 23 (10.0%) and unclassified (penta negative) 99 (42.8%). The cumulative hybrid category comprised 23 (10.0%). It included four subtypes, LBHH 8 (3.5%), LBBH 9 (3.9%), HBH 3 (1.3%), and LABH 3 (1.3%) (see Table 2).

**Table 2:** Prevalence of molecular classes.

	Number	%
LUMA	9	3.9

LUMB	37	16.0

HER2	40	17.3

Basal	23	10.0

UC (penta -ve)	99	42.8

Hybrid	23	10.0

LBHH	8	3.5

LABH	3	1.3

HBH	3	1.3

LBBH	9	3.9

Total	231	100

LUMA = luminal A, LUMB = luminal B, HER2 = human epidermal growth factor receptor 2, UC = unclassified, LBHH = luminal B-HER2 hybrid, LABH = luminal A-basal hybrid, HBH = HER2-basal hybrid, LBBH = luminal B-basal hybrid.

**Figure 2:**
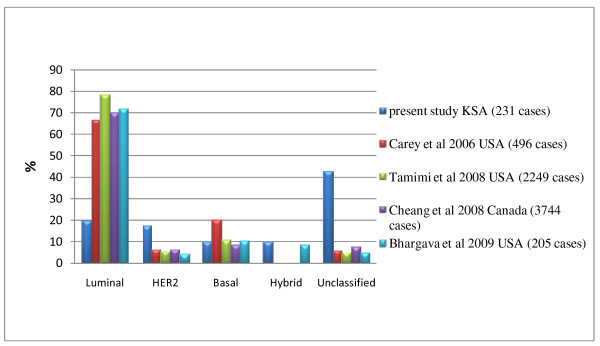
**Prevalence of Molecular types of breast cancer compared to 4 large Western studies**.

**Figure 3:**
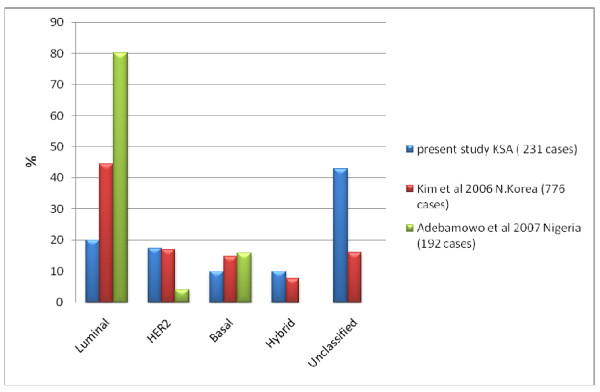
**Prevalence of Molecular types of breast cancer compared to 2 large Asian and African studies**.

Comparing the prevalence patterns in our study with that of Western [17,18,20,26] and other regionally based studies [27,28] revealed striking differences (see Figures 2 and 3). The main differences were in the luminal and the unclassified groups. Luminal (HR +ve) tumors as a group had a low prevalence in our cases (19.9%), in contrast to its high prevalence as reported in the Western studies: 72% (Bhargava *et al. *[20] ), 66.9% (Carey *et al. *[17] ), 70.28% (Cheang *et al. *[26] ), 78.6% (Tamimi *et al. *[18] ), and in regionally based studies from N Korea, 44.5% (Kim *et al. *[27] ) and Nigeria, 80.2% (Adebamowo *et al. *[28] ). In addition, LUMB (16%) was more prevalent than LUMA (3.9%) in our study while in those other studies LUMA was the more prevalent (ranging from 39.9 - 77.6%). Realizing that discrepancies could arise because of the different methods used in the differentiation between LUMA and LUMB, we grouped them together as luminal tumors for comparison studies. The unclassified tumors represented a small group in the studies from other regions, whereas they constituted a large proportion of our cases. The prevalence of HER2 (17.31%) in our study was also higher than that of the compared studies, with a range of 4 - 6.6%.

A similar prevalence pattern with a still significantly low incidence of luminal tumors (p = < 0.001 ) was obtained when molecular classification was attempted using other criteria previously described by several groups [17,18,20]. Analysis with hybrid "luminal-HER2" and "HER2-basal" cases included among luminal and HER2 classes, respectively, yielded the following results: Luminal 66 cases (28.5%), HER2 43 (18.6%), Basal 23 (10%) and Unclassified (penta negative) 99 (42.8%). However, the prevalence of LUMA increased from 3.9% to 25.1% (58 cases), while LUMB dropped from 16% to 3.4% (8 cases). Yet the prevalence of LUMA was still significantly lower than that reported in the above mentioned studies (p = < 0.001).

### Carcinoma types & grades

In this study, invasive ductal carcinoma ( not otherwise specified) was the predominant type, comprising 183 (79.2%) of cases, followed by invasive lobular carcinoma 9 (3.89%) of cases. *In situ *carcinoma comprised 15 (6.49%) and a miscellaneous group of other rare tumors (medullary carcinoma, apocrine carcinoma, mixed ductal and lobular carcinoma, juvenile secretory carcinoma) 24 cases (10.38%). The tumors were predominantly grade 2, 104 cases (51.2%), followed by grade 3, 89 (43.8%). Grade 1 was rare, comprising only 10 (4.9%) of our cases. In total, 203 cases were graded. The remaining were invasive lobular carcinomas and *in situ *ductal carcinomas for which the Bloom Scarf Richardson grading system is not applicable. A strong correlation was noted between invasive lobular carcinoma and hormone receptor expression with 8 out of 9 lobular carcinoma cases (88.9%) classifiable as luminal cancers. Otherwise, there was no association between the molecular class and the histologic type or grade of the tumor (see Tables 3 &4).

**Table 3:** Correlation of molecular class with histological type of tumor.

	LUMA No.(%)	LUMB No(%)	HER2 No(%)	Basal No(%)	Hybrid No(%)	UC N0(%)	Total No(%)
IDC	6 (3.3)	27 (14.8)	29 (15.8)	20 (10.9)	16 (8.7)	85 (46.4)	183 (79.2)

ILC	3 (33.3)	5 (55.6)	0 (0)	0 (0)	0 (0)	1 (11.1)	9 (3.89)

ISC	0 (0)	3 (20.)	7 (46.7)	0 (0)	3 (20)	2 (13.3)	15 (6.49)

Other	0 (0)	2 (8.3)	4 (16.7)	3 (12.5)	4 (16.7)	11(45.8)	24 (10.38)

IDC = Invasive ductal carcinoma, ILC = Invasive lobular carcinoma, ISC = In situ carcinoma, LUMA = luminal A, LUMB = luminal B, HER2 = human epidermal growth factor receptor 2, UC = unclassified

**Table 4:** Correlation of molecular class with grade of tumors.

	LUMA No (%)	LUMB No (%)	HER2 No (%)	Basal No (%)	Hybrid No (%)	UC No (%)	Total No (%)
grade 1	2 (20)	3 (30)	0 (0)	2 (20)	1 (10)	2 (20)	10 (4.9)

grade 2	5 (4.3)	16(15.3)	13 (12.5)	15 (14.5)	13 (12.5)	42 (40.5)	104 (51.23)

grade 3	3 (3.4)	5 (5.6)	14 (15.7)	11 (12.3)	14 (15.7)	42 (47)	89 (43.8)

LUMA = luminal A, LUMB = luminal B, HER2 = human epidermal growth factor receptor 2, UC = unclassifiable

### Ki-67 Proliferation Index

Table 5 shows the relative prevalence of various molecular classes of tumors with a moderate to high Ki67 proliferation index. The highest prevalence was noted in the basal class (78.3% of cases), followed by hybrid (47.8%), then HER2 (47.5%), LUMB (43.24%), UC (35.35%) and LUMA (22.2%) classes. However, a significantly higher value was noted only in the basal and a significantly lower value was seen only in the LUMA (p = 0.007). In addition, the mean Ki-67 proliferation index was significantly higher only in the basal compared to all others, whereas the mean values were comparable for the other types (42.82 in the basal class vs.14.48-16 in the others, P = 0.003) (see Table 6). Comparing the Ki-67 index of the hybrid cases with their constituents revealed that in LBBH, the incidence of moderate to high Ki-67 indices was lower than in both the LUMB and basal classes, but it was closer to that of LUMB (Table 7). In LBHH, the incidence was significantly higher than HER2 and LUMB (p = 0.04; see Table 8).

**Table 5:** Correlation of molecular class with Ki-67 and p53.

	Cases with moderate to high Ki67 index		Cases with focal to diffusely positive p53	
	**No**	**%**	**No**	**%**

LUMA	2	22.2	0	0

LUMB	15	43.24	5	13.5

HER2	19	47.5	18	45

Basal	18	78.3	7	30.4

Hybrid	11	47.8	4	17.4

Unclassified	35	35.35	16	16.2

LUMA = luminal A, LUMB = luminal B, HER2 = human epidermal growth factor receptor 2

**Table 6:** Mean Ki67 index of molecular classes.

Class	Mean Ki-67 index
LUMA	16

LUMB	14.48

HER2	14.62

Basal	42.82

Hybrid	16.78

UC/penta-ve	15.83

LUMA = luminal A, LUMB = luminal B, HER2 = human epidermal growth factor receptor 2, UC = unclassified.

**Table 7:** LUMB-Basal hybrid (LBBH) compared to LUMB and Basal.

	Cases with moderate to high Ki-67 index		Cases with focal to diffusely positive p53	
	No	%	No	%

LBBH	3	30	1	10

LUMB	15	43.24	5	13.5

Basal	18	78.3	7	30.4

LBBH = luminal B-basal hybrid, LUMB = luminal B

**Table 8:** LUMB-HER2 hybrid (LBHH) compared to LUMB and HER2.

	Cases with moderate to high Ki67 index		Cases with focal to diffusely positive p53	
	**No**	**%**	**No**	**%**

LBHH	5	62.5	1	12.5

LUMB	15	43.24	5	13.5

HER2	19	47.5	18	45

LBHH = luminal B-HER2 hybrid, LUMB = luminal B, HER2 = human epidermal growth factor receptor 2

### p53 over-expression

Focal or diffuse positivity for p53 was noted in 50 out of the 231 (21.64%) breast cancer cases. The prevalence of positivity for p53 is shown in Table 5. It was highest in the HER2 (45% of cases), followed by the basal (30.4%), then the hybrid (17.4%), UC (16.2%), and LUMB (13.5%) classes. The level for HER2 was significantly higher than the others (p = 0.001). p53 over-expression was not encountered in the LUMA type. Comparing the p53 positivity of hybrid cases revealed that in LBBH the incidence of focal to diffuse positivity was lower than in both LUMB and basal classes but it was closer to LUMB (see Table 7). In LBHH, the incidence was almost equal to LUMB, and significantly lower than HER2 (p < 0.001; see Table 8).

## Discussion

In this study we have demonstrated that by using IHC as a surrogate for gene expression profiling, there is a unique molecular class prevalence pattern in our population, a relative paucity of luminals and preponderance of unclassified cases being its highlighting features. In addition, other than a strong correlation between invasive lobular carcinoma and hormone receptor expression, there was no association between molecular classes and the histologic type of the cancer. This perfectly matches with the general belief that differences in prognosis and response to treatment within morphologically identical tumors can largely be attributed to genetic/molecular differences [6]. It also emphasizes a justifiable need for replacing current morphology based classification systems of breast cancer by a molecular classification that may be more relevant prognostically [7].

Several issues in our study need to be discussed. The unclassified category in our study comprised 42.86% of cases, which is significantly higher than in studies by Carey *et al. *(6.25%) [17], Tamimi *et al. *(4.9%) [18], Cheang *et al. *(8%) [26], and Bhargava *et al. *(4.87%) [20] (p = < 0.001). It is also significantly higher than that reported by Kim *et al. *[27] in Korean women (15.9%, p = < 0.001). Adebamowo *et al.*, in their study on Nigerean women [28] did not report any cases in the unclassified category. The presence of a dominant unclassified category merits further investigation to gain further insight into its nature. It highlights the inability of currently used molecular analytic spectrum to adequately represent all the molecular types and stresses on the immense need for the development of new prognostic and predictive markers in breast cancer diagnosis, as has also been suggested by Brennan *et al. *[29].

Unclassified cases were initially considered to be synonymous with '*normal-like' *breast cancers. These tumors cluster with non-tumoral breast cancer cells and exhibit over-expression of PIK3R1 and AKR1C1, in addition to other genomic alterations [30]. The current concept states that the *'normal-like' *subtype is absolutely different from the unclassified (penta negative) "ER-, PR-, HER2-, CK5/6, and EGFR-" group, as absent or decreased expression of basal markers is not a feature compatible with the '*normal-like' *molecular class [31]. They are very good prognostically [30] and are grouped with the luminals, both of which exhibit low pathologic complete remission rates of 6% [32].

According to Huo *et al.*, the unclassified category comprises two contrasting branches, a bad prognostic branch, characterized by the expression of vascular endothelial growth factor, B-cell lymphoma extra-large protein, and Cyclin E, and the a good prognosis branch, characterized by expression of B-cell lymphoma protein 2 and Cyclin D1 as its distinguishing features [33]. The unclassified and '*normal-like' *are completely separate entities and IHC surrogates for these categories have not yet been developed. Associating these with a particular set of negative or absent markers may lead to misinterpretations of their intrinsic biological characteristics [33].

In this study 35.35% of the unclassified tumors exhibited a moderate to high Ki-67 proliferation index, and 16.1% demonstrated p53 mutations, suggesting the presence of two divergent patterns of unclassified tumors in our population. This hypothesis has not yet been validated, but further analysis of this finding is essential.

Hormone receptor positive tumors are usually diagnosed irrespective of stratification of receptor staining intensity or distinction between ER and PR positivity. Studies demonstrate that the benefit of endocrine therapy is directly related to estrogen receptor levels [34], with responses beginning in tumors with > 10% positive nuclei, and increasing gradually with increased receptor expression [35]. Strong ER+ cases benefit from endocrine therapy alone, in contrast to those with low to moderate ER positivity ( by Alred score) that demonstrate a better outcome with the addition of chemotherapy [36]. Therefore, the practice of assigning all strata of ER positivity to one group should be revised.

The prognostic significance of PR presence, in ER + tumors, with regards to endocrine (tamoxifen) treatment is controversial [37]. Studies suggest that PR has no prognostic predictive role in these cases [34,38]. The level of PR positivity is an independent prognostic marker, with Stendahl *et al. *demonstrating that the response to tamoxifen begins to be seen in tumors with more than 75% positive nuclei [35]. With these separate prognostic implications, the concept of a conjoined ER/PR group also merits re-evaluation.

Luminal tumors in our study comprised 19.9% of all cases, again, a prevalence pattern that is unique to our population. This is in stark contrast to the prevalence pattern seen in others, specifically, the more profiled Caucasian populations [17,18,20,26], as well as the populations from regional based studies in Korea [27] and Nigeria [28]. LUMA represented merely 3.9% of all cases, which is significantly less than those reported in the other studies, which ranged from 54%-77.6% (p = < 0.001). Analysis with hybrid "luminal-HER2" and "HER2-basal" cases included among luminal and HER2 classes, respectively [17,18,20] still yielded a significantly low prevalence of luminal tumors including LUMA in our population. According to studies in Western populations, Luminal-A tumors are often the most prevalent and have a relatively good prognosis. Adelaide *et al. *[39] showed luminal-A class to be characterized by a gain of 1q, 8p12-ter, loss of 1p36-ter, and an amplification in 5q35, along with other genetic alterations [30]. It is possible that the low representation of luminal tumors, specifically the LUMA, seen in our population may be reflective of a different set of genetic alterations. This possibility requires investigation, but such queries provide the framework for future research.

LUMB breast cancers are characterized by expression of HR along with HER2 associated genes (i.e., ERBB2 and GRB7) and a cell proliferation pattern designated by expression of MK167, CCNBI and MYBL2 [26]. They are associated with a poorer clinical outcome than LUMA tumors. Identification of luminal B tumors at the protein level is a point of controversy. Some authors have used the co-expression of HR and HER2 to define this group, based on the fact that the HER2 associated genes (i.e ERBB2 and GRB7) are expressed in 30-50% of LUMB tumors, and realizing that this will not identify all of the tumors in this category, with a number of them ultimately being classified as LUMA tumors [17]. Bhargava *et al. *defined LUMA and LUMB as pure hormone receptor positive, the differentiating feature between them being the intensity of receptor positivity [20]. Cheang *et al. *added that a Ki67 proliferation index of more than 13.25% is a hallmark of LUMB tumors [26]. Ki67 is a nuclear marker of cell proliferation, and its expression correlates proportionally to poorer clinical outcomes [40-42]. Although it is not currently included in routine clinical decisions, recent studies [43,44], have indicated that alterations in its levels after neo-adjuvant endocrine treatment simultaneously alters the long-term patient outcome. As suggested from gene expression profiling, only 30% of LUMB tumors are HER2 positive, indicating that this clinical marker is not sensitive enough to identify most LUMB breast cancers [26]. These tumors are also endocrine (tamoxifen) resistant and require estrogen deprivation in addition to blockage of HER2 pathways [34]. Therefore, including them as an integral component of endocrine sensitive tumors may not be justified. Due to these complexities, the HER2+ tumors need to be considered separately from pure luminal tumors, which should be further categorized as luminal A and luminal B, with those showing co-positivity of HER2 grouped into a separate hybrid category Luminal-HER2 hybrids [20].

The prevalence of LUMB in our study (16%) was similar to those of other studies [17,18,20]. The only study with which this group showed a striking disparity was that of Cheang *et al. *(16 vs. 32%) [26]. The reason may be that they have used a Ki-67 proliferation index of 13.25% as a cutoff between LUMA and LUMB, as compared with no cutoff used by us. They demonstrated that although gene expression profiling remains the most sensitive method, Ki-67 proliferation added to ER, PR, and HER2 status can be used to identify additional LUMB tumors that would not otherwise be identified by these three markers alone [26]. This may have led to increased expression of LUMB in their study. Ki-67 is a well established proliferation marker in cancer and an excellent biomarker for LUMB tumors. Ki-67 assessment has been a matter of controversy as some studies have used 10% [45,46] or 20% [47,48] as cutoff points, while others reported use of values around the mean [49] or median [50,51]. In our study we did not use a cutoff point but used 10% as a starting point for moderate proliferative activity and demonstrated that 22.2% of LUMA and 43.24% of LUMB tumors had a moderate to high Ki-67 labeling index. As a matter of fact, LUMA tumors showed the least prevalence of moderate to high Ki-67 proliferation index in our study. This is in agreement with Bhargava *et al *[20] who reported the lowest average Ki-67 labeling index in their LUMA group.

The prevalence of the HER2+ class in our study was almost identical to that observed by Kim *et al. *[27] in North Korean women (17.31% vs 17.1%). However, it was significantly higher than the prevalence reported in each of the other five studies that we used for the comparison (17.31% vs. a mean of 5.33%) (p = <0.001) [17,18,20,26,28]. This difference re-emphasizes that there are regional variations in prevalence, a concept further supported Al-Kuraya *et al. *who demonstrated that a markedly higher frequency of HER2 and MYC amplifications are seen in Saudi as compared to Swiss breast cancers [2].

HER2 over-expression is prognostically unfavorable but is associated with better responsiveness to trastuzumab (Herceptin) therapy and anthracycline-based chemotherapy [45]. Thus its accurate analysis and precise quantification is essential [46] to identify patients who are eligible for trastuzumab therapy [47].

Studies have demonstrated that the most common mechanism (90-96%) of HER2 over-expression is by gene amplification [45,48-50]. Both IHC and FISH have been validated as methods for its analysis [46]. There has recently been the introduction of bright field *in situ *hybridization techniques such as chromogenic *in situ *hybridization (CISH) and silver enhanced *in situ *hybridization (SISH). Here, a peroxidase enzyme labeled probe with chromogenic detection is used instead of a fluorescent labeled probe, hence the results can be visualized by standard bright field microscopy. Advantages over FISH are that simultaneous histological and HER2 assessment can be carried out; moreover the signals do not decay over time [46]. We employed SISH for confirmation of the HER2 positivity on all our cases.

HER2 positivity in our study was seen in two sets of cases, exclusive HER2 positive cases and HER2 positivity in association with luminal and basal markers as part of the spectrum of the hybrid class. Bull *et al. *have demonstrated increased frequency of p53 mutations in carcinomas with neu/erbB2 amplifications [51]. In our study we also observed that 45% of HER2+ tumors demonstrated focal or diffuse positivity, thus making HER2 the major class exhibiting p53 mutations. This level for HER2 was significantly higher than the other types (p = 0.001). The combination of HER2 and p53 mutations is associated with an increased risk of disease recurrence and overall mortality in comparison with patients who have only one, or neither, of the mutations [51]. p53 over-expression was not encountered in the LUMA class which is consistent with the fact that p53 over-expression is known to be associated with high proliferation rates and a poor clinical outcome [52].

In addition to HER2 positive LUMB cases categorized as LBHH, we also encountered LABH, LBBH and HBH cases (Table 2). In an attempt to define which of the molecular classes these cases may be most similar to, we compared the Ki-67 proliferative index and p53 expression levels in LUMB-basal hybrid and LUMB-HER2 hybrid cases to those of LUMB, basal, and HER2 classes. In this context, LUMB-HER2 hybrid cases seem to represent a biological overlap between its two constituent classes. Their p53 expression appeared at par with that of LUMB but was significantly lower than that of HER2. On the other hand, their Ki67 proliferation index (moderate to high) was significantly higher than that of both the HER2 and the LUMB molecular classes. This varied, distinctive pattern merits them an attribution of a separate molecular class rather than a subtype of the luminal class as conceived by several authors [17,18]. In this we agree with Bhargava *et al. *[20]. On the other hand, LUMB-basal hybrid cases seem to be closer to the LUMB class. Consequently, they may be considered as part of a "spectrum" of the LUMB class rather than a separate subtype. This is again in agreement with Bhargava *et al. *[20]. A similar analysis was not possible for LUMA-basal and HER2-basal hybrid cases because of their limited number (3 cases each). These preliminary observations, however, need to be validated through clinical investigation of a large number of patients. But it will still be important to determine if this grouping will be valid therapeutically, and whether the co-existence of basal markers in luminal and HER2 categories should become the basis for a different treatment approach.

The basal category, with a frequency of 9.95%, represented a pattern harmonious with Bhargava *et al. *(10.7%) [20], Cheang *et al. *(9%) [26], and Tamimi *et al.*(10.9%) [18]. The only study with a higher basal prevalence was Carey *et al.*, with 20.1% [17]. Tan *et al *[53] demonstrated that increased levels of Ki 67 show a correlation with immuno-negativity for estrogen receptors, high tumor grade and increased mitotic activity, while in another study Kuroda *et al *[54] showed high levels of Ki-67, p-53 and P-glycoprotein more in basal than in non basal types of breast cancer. In our study too the Ki-67 index was highest in the basal type followed by HER2, hybrids, LUMB, UC and finally LUMA. The significantly high Ki67 proliferation index in our basal-like class of breast cancer is in agreement with Carey *et al *[17], Siziopikou & Colbeigh [55] and Bhargava *et al *[20].

Triple negative breast cancer is a type of aggressive breast cancer lacking the expression of ER, PR and HER2. It was found to be much more frequent in BRCA 1 and BCRA 2 positive than in BRCA-negative patients by Atchley *et al *[56]. The currently available ER-targeted and HER-2-based therapies are not effective for treating triple negative breast cancer. Recent studies have revealed a number of novel molecular features of triple negative breast cancer. Gaining better insights into these molecular pathways may lead to identification of novel biomarkers and targets for development of diagnostic and therapeutic approaches for its prevention and treatment [57]. The term "triple negative" is a term frequently used, sometimes interchangeably with basal type, by both researchers and clinicians. It is now accepted that not all triple negative cancers are basal [58] and that they should not be taken as synonymous. There is an overlap with the basal group in 60-90% of cases and they are a component of the unclassified penta negative type. Triple negative tumors not exhibiting basal markers are the unclassified or the penta negative group in our study.

## Conclusions

We demonstrated that ethnic variations may exist in molecular class prevalence pattern. A distinctive, indigenous distribution pattern was exhibited that was in stark contrast with that seen in all the western studies. The inadequacy of the currently used molecular analytic spectrum was also highlighted as a significant fraction of our cases failed to express the IHC surrogate markers for the classified molecular entities. This fact stresses on the urgent need for further refinement and development of the existing classification systems. Expression of novel combinations of molecular classes forming various sub-classes were demonstrated in the newly emerging hybrid category of tumors. This could, in the future alter and modify the course of therapeutic strategies, that are currently more class specific.

Our knowledge about breast cancer molecular classes has increased tremendously, but beyond a certain limit, there are queries and dilemmas that still remain unanswered, and which remain important for future studies.

## Competing interests

The authors declare that they have no competing interests.

## Authors' contributions

DT, MS, and AA, AO were responsible for the design of the study, selection of the cases, collection of information, and TMA. DT, MS, AA were also responsible for IHC and SISH staining and interpretation of the results. DT and MS were evaluating and reporting the results of the stains. DT, MS, AA were involved in the writing up of the manuscript. AH helped in the statistical analysis. All authors have read and approved the final manuscript.

## Pre-publication history

The pre-publication history for this paper can be accessed here:

http://www.biomedcentral.com/1471-2407/10/223/prepub

## References

[B1] Tarone RE Breast cancer trends among young women in the United States Epidemiology 2006 17 5 588 590 10.1097/01.ede.0000229195.98786.ee 16804474

[B2] Al-Kuraya K Schramel P Skeik Amr S Torhorst J Tapia C Novotny H Spichtin H Maurer R Mirlacher M Simon R Sauter G Predominance of high grade pathway in breast cancer development of middle east Modern Pathology 2005 18 891 897 10.1038/modpathol.3800408 15803183

[B3] Registry NC Cancer Incidence Report Saudi Arabia 2002 Riyadh 2007

[B4] Ibrahim EM al-Mulhim FA al-Amri A al-Muhanna FA Ezzat AA Stuart RK Ajarim D Breast cancer in the Eastern province of Saudi Arabia Med Oncol 1998 15 4 241 247 10.1007/BF02787207 9951687

[B5] Ezzat AA Ibrahim EM Raja MA Al-Sobhi S Rostom A Stuart RK Locally advanced breast cancer in Saudi Arabia: high frequency of stage III in a young population Med Oncology 1999 16 2 95 103 10.1007/BF02785842 10456657

[B6] Pusztai L Cristofanilli M Paik S New generation of molecular prognostic and predictive tests for breast cancer Semin Oncol 2007 34 2 Suppl 3 S10 6 10.1053/j.seminoncol.2007.03.015 17512431

[B7] Pusztai L Mazouni C Anderson K Wu Y Symmans WF Molecular classification of breast cancer: limitations and potential Oncologist 2006 11 8 868 877 10.1634/theoncologist.11-8-868 16951390

[B8] Wood WCMH Solin LJ Olopade OI Malignant tumors of the breast 2005 7 Philadelphia: Lippincott Wiliams & Wilkins

[B9] Bast RC Jr Ravdin P Hayes DF Bates S Fritsche H Jr Jessup JM Kemeny N Locker GY Mennel RG Somerfield MR Update of recommendations for the use of tumor markers in breast and colorectal cancer: clinical practice guidelines of the American Society of Clinical Oncology J Clin Oncol 2001 19 1865 1878 11251019 10.1200/JCO.2001.19.6.1865

[B10] Perou CM Sorlie T Eisen MB Rijn M van de Jeffrey SS Rees CA Pollack JR Ross DT Johnsen H Akslen LA Fluge O Pergamenschikov A Williams C Zhu SX Lønning PE Børresen-Dale AL Brown PO Botstein D Molecular portraits of human breast tumours Nature 2000 406 747 752 10.1038/35021093 10963602

[B11] Pusztai L Ayers M Stec J Clark E Hess K Stivers D Damokosh A Sneige N Buchholz TA Esteva FJ Arun B Cristofanilli M Booser D Rosales M Valero V Adams C Hortobagyi GN Symmans WF Gene expression profiles obtained from fine-needle aspirations of breast cancer reliably identify routine prognostic markers and reveal large-scale molecular differences between estrogen-negative and estrogen-positive tumors Clin Cancer Res 2003 9 2406 2415 12855612

[B12] Sorlie T Tibshirani R Parker J Hastie T Marron JS Nobel A Deng S Johnsen H Pesich R Geisler S Demeter J Perou CM Lønning PE Brown PO Børresen-Dale AL Botstein D Repeated observation of breast tumor sub types in independent gene expression data sets Proc Natl Acad Sci USA 2003 100 8418 8423 10.1073/pnas.0932692100 12829800 PMC166244

[B13] Sotiriou C Neo SY McShane LM Korn EL Long PM Jazaeri A Martiat P Fox SB Harris AL Liu ET Breast cancer classification and prognosis based on gene expression profiles from a population-based study Proc Natl Acad Sci USA 2003 100 10393 10398 10.1073/pnas.1732912100 12917485 PMC193572

[B14] Nielsen TO Hsu FD Jensen K Cheang M Karaca G Hu Z Hernandez-Boussard T Livasy C Cowan D Dressler L Akslen LA Ragaz J Gown AM Gilks CB Rijn M van de Perou CM Immunohistochemical and clinicalcharacterization of the basal-like subtype of invasive breast carcinoma Clin Cancer Res 2004 10 5367 5374 10.1158/1078-0432.CCR-04-0220 15328174

[B15] Abd El-Rehim DM Ball G Pinder SE Rakha E Paish C Robertson JF Macmillan D Blamey RW Ellis IO Highthroughput protein expression analysis using tissue microarray technology of a large well-characterised series identifies biologically distinct classes of breast cancer confirming recent cDNA expression analyses Int J Cancer 2005 116 340 350 10.1002/ijc.21004 15818618

[B16] Jacquemier J Ginestier C Rougemont J Bardou VJ Charafe-Jauffret E Geneix J Adelaide J Koki A Houvenaeghel G Hassoun J Maraninchi D Viens P Birnbaum D Bertucci F Protein expression profiling identifies subclasses of breast cancer and predicts prognosis Cancer Res 2005 65 767 779 15705873

[B17] Carey LA Perou CM Livasy CA Dressler LG Cowan D Conway K Karaca G Troester MA Tse CK Edmiston S Deming SL Geradts J Cheang MC Nielsen TO Moorman PG Earp HS Millikan RC Race, breast cancer subtypes, and survival in the Carolina Breast Cancer Study JAMA 2006 295 2492 2502 10.1001/jama.295.21.2492 16757721

[B18] Tamimi RM Baer HJ Marotti J Galan M Galaburda L Fu Y Deitz AC Connolly JL Schnitt SJ Colditz GA Collins LC Comparison of molecular phenotypes of ductal carcinoma in situ and invasive breast cancer Breast Cancer Research 2008 10 R67 10.1186/bcr2128 18681955 PMC2575540

[B19] Al Tamimi DM Bernard PS Shawarby MA Al-Amri AM Hadi MA Distribution of molecular breast cancer subtypes in Middle Eastern-Saudi Arabian women - A pilot study Ultrastructural Pathology 2009 33 141 150 19728229 10.1080/01913120903183135

[B20] Bhargava R Striebel J Beriwal S Flickinger JC Onisko A Ahrendt G Dabbs DJ Prevalence, Morphologic Features and Proliferation Indices of Breast Carcinoma Molecular Classes Using Immunohistochemical Surrogate Markers Int J clin Exp Pathol 2009 444 455 19294003 PMC2655155

[B21] Wolff AC Hammond ME Schwartz JN Hagerty KL Allred DC Cote RJ Dowsett M Fitzgibbons PL Hanna WM Langer A McShane LM Paik S Pegram MD Perez EA Press MF Rhodes A Sturgeon C Taube SE Tubbs R Vance GH Vijver M van de Wheeler TM Hayes DF American Society of Clinical Oncology/College of American Pathologists Guideline Recommendations for Human Epidermal Growth Factor Receptor 2 Testing in Breast Cancer Arch Pathol Lab Med 2007 31 1 18 10.5858/2007-131-18-ASOCCO 19548375

[B22] Bhargava R Gerald WL Li AR Pan Q Lal P Ladanyi M Chen B EGFR gene amplification in breast cancer: correlation with epidermal growth factor receptor mRNA and protein expression and HER-2 status and absence of EGFR-activating mutations Modern Pathology 2005 18 1027 1033 10.1038/modpathol.3800438 15920544

[B23] Foulkes WD Stefansson IM Chappui PO Bégi LR Goffin JR Won N Trude M Akslen LA Germline BRCA1 Mutations and a Basal Epithelial Phenotype in Breast Cancer Journal of the National Cancer Institute 2003 95 19 1482 1485 14519755 10.1093/jnci/djg050

[B24] De Manzoni G Verlato G Tomezzoli A Study of Ki 67 immunoreactivity as a prognostic indicator in patients with advanced gastric cancer Jpn J Clin Oncol 1998 28 534 537 10.1093/jjco/28.9.534 9793024

[B25] Kleer CG Giordano TJ Braun T Oberman HA Pathologic, Immunohistochemical, and Molecular Features of Benign and Malignant Phyllodes Tumors of the Breast Mod Pathol 2000 14 3 185 190 10.1038/modpathol.3880282 11266524

[B26] Cheang MCU Chia SK Voduc D Gao D Leung S Snider J Watson M Davies S Bernard PS Parker JS Perou CM Ellis MJ Nielsen TO Ki67 Index, HER2 Status, and Prognosis of Patients With Luminal B Breast Cancer J Natl Cancer Inst 2009 101 736 750 10.1093/jnci/djp082 19436038 PMC2684553

[B27] Kim MJ Ro JY Ahn SH Kim HH Kim SB Gong G Clinicopathologic significance of the basal-like subtype of breast cancer: a comparison with hormone receptor and HER2/neu-overexpressing phenotypes Hum Pathol 2006 37 9 1217 1226 10.1016/j.humpath.2006.04.015 16938528

[B28] Adebamowo CA Famooto A Ogundiran TO Aniagwu T Nkwodimmah C Akang EE Immunohistochemical and molecular subtypes of breast cancer in Nigeria Breast cancer research and treatment 2007 110 1 183 188 10.1007/s10549-007-9694-5 17674190

[B29] Brennan DJ Gallagher WM Prognostic ability of a panel of immunohistochemistry markers - retailoring of an 'old solution' Breast Cancer Res 2008 10 1 102 10.1186/bcr1854 18331621 PMC2374964

[B30] Guedj M A synthetic review of the five molecular Sorlie's subtypes in breast cancer http://mickael.guedj.googlepages.com/mguedjCITReview01.pdf

[B31] Yu KD Shen ZZ Shao ZM The immunohistochemically "ER-negative, PR-negative, HER2-negative, CK5/6-negative, and HER1-negative" subgroup is not a surrogate for the normal-like subtype in breast cancer Breast Cancer Res Treat 2009 118 3 661 663 10.1007/s10549-009-0522-y 19714461

[B32] Rouzier R Perou CM Symmans WF Ibrahim N Cristofanilli M Anderson K Breast cancer molecular subtypes respond differently to preoperative chemotherapy Clin Cancer Res 2005 11 16 5678 5685 10.1158/1078-0432.CCR-04-2421 16115903

[B33] Huo D Ikpatt F Khramtsov A Dangou JM Nanda R Dignam J Zhang B Grushko T Zhang C Oluwasola O Malaka D Malami S Odetunde A Adeoye AO Iyare F Falusi A Perou CM Olopade OI Population differences in breast cancer: survey in indigenous African women reveals over-representation of triple-negative breast cancer J Clin Oncol 2009 27 27 4515 4521 Epub 2009 Aug 24 10.1200/JCO.2008.19.6873 19704069 PMC2754904

[B34] Rastelli F Crispino S Factors predictive of response to hormone therapy in breast cancer Tumori 2008 94 3 370 383 18705406 10.1177/030089160809400314

[B35] Stendahl M Rydén L Nordenskjöld B Jönsson PE Landberg G Jirström K High progesterone receptor expression correlates to the effect of adjuvant tamoxifen in premenopausal breast cancer patients Clin Cancer Res 2006 12 15 4614 4618 10.1158/1078-0432.CCR-06-0248 16899609

[B36] Albain K Barlow W O'Malley F Siziopikou k Yeh IT Ravdin P Lew D Farrar W Burton G Ketchel S for the breast cancer intergroup of North America Concurrent (CAFT) versus sequential (CAF-T) chemohormonal therapy (cyclophosphamide, doxorubicin, 5-fluorouracil, tamoxifen) versus T alone for postmenopausal, estrogen (ER) and/or progesterone (PR) receptor-positive breast cancer: Mature outcomes and new biologic correlates on phase III intergroup trial 0100 (SWOG-8814) Breast Cancer Res Treat 2004 88 S20 (abstract 37)

[B37] Ma CX Sanchez CG Ellis MJ Predicting endocrine therapy responsiveness in breast cancer Oncology 2009 2 133 142 19323294

[B38] Dowsett M Houghton J Iden C Salter J Farndon J A'Hern R Sainsbury R Baum M Benefit from adjuvant tamoxifen therapy in primary breast cancer patients according oestrogen receptor, progesterone receptor, EGF receptor and HER2 status Ann Oncol 2006 17 5 818 826 10.1093/annonc/mdl016 16497822

[B39] Adelaide J Integrated profiling of basal and luminal breast cancers Cancer Research 2007 7 231 10.1158/0008-5472.CAN-07-2536 18089785

[B40] Domagala W Markiewski M Harezga B Dukowicz A Osborn M Prognostic signifi cance of tumor cell proliferation rate as determined by the MIB-1 antibody in breast carcinoma: its relationship with vimentin and p53 protein Clin Cancer Res 1996 2 1 147 154 9816101

[B41] Trihia H Murray S Price K Gelber RD Golouh R Goldhirsch A Coates AS Collins J Castiglione-Gertsch M Gusterson BA International Breast Cancer Study Group Ki-67 expression in breast carcinoma: its association with grading systems, clinical parameters, and other prognostic factors -- a surrogate marker? Cancer 2003 97 5 1321 1331 10.1002/cncr.11188 12599241

[B42] de Azambuja E Cardoso F de Castro G Jr Colozza M Mano MS Durbecq V Sotiriou C Larsimont D Piccart-Gebhart MJ Paesmans M Ki-67 as prognostic marker in early breast cancer: a meta-analysis of published studies involving 12,155 patients Br J Cancer 2007 96 10 1504 1513 10.1038/sj.bjc.6603756 17453008 PMC2359936

[B43] Ellis MJ Coop A Singh B Tao Y Llombart-Cussac A Jänicke F Mauriac L Quebe-Fehling E Chaudri-Ross HA Evans DB Miller WR Letrozole inhibits tumor proliferation more effectively than tamoxifen independent of HER1/2 expression status Cancer Res 2003 63 19 6523 6531 14559846

[B44] Dowsett M Smith IE Ebbs SR Dixon JM Skene A A'Hern R Salter J Detre S Hills M Walsh G IMPACT Trialists Group Prognostic value of Ki67 expression after short-term presurgical endocrine therapy for primary breast cancer J Natl Cancer Inst 2007 99 2 167 170 10.1093/jnci/djk020 17228000

[B45] Keshgegian AA Cnaan A Proliferation markers in breast carcinoma. Mitotic figure count, S-phase fraction, proliferating cell nuclear antigen, Ki-67 and MIB-1 Am J Clin Pathol 1995 104 1 42 49 7611179 10.1093/ajcp/104.1.42

[B46] Bevilacqua P Verderio P Barbareschi M Bonoldi E Boracchi P Dalla Palma P Gasparini G Lack of prognostic significance of the monoclonal antibody Ki-S1, a novel marker of proliferative activity, in node-negative breast carcinoma Breast Cancer Res Treat 1996 37 2 123 133 10.1007/BF01806494 8750580

[B47] Clahsen PC Velde CJ van de Duval C Pallud C Mandard AM Delobelle-Deroide A Broek L van den Vijver MJ van de The utility of mitotic index, oestrogen receptor and Ki-67 measurements in the creation of novel prognostic indices for node-negative breast cancer Eur J Surg Oncol 1999 25 4 356 363 10.1053/ejso.1999.0657 10419704

[B48] Joensuu H Isola J Lundin M Salminen T Holli K Kataja V Pylkkänen L Turpeenniemi-Hujanen T von Smitten K Lundin J Amplification of erbB2 and erbB2 expression are superior to estrogen receptor status as risk factors for distant recurrence in pT1N0M0 breast cancer: a nationwide population based study Clin Cancer Res 2003 9 3 923 930 12631589

[B49] Goodson WH Moore DH Ljung BM Chew K Mayall B Smith HS Waldman FM The prognostic value of proliferation indices: a study with in vivo bromodeoxyuridine and Ki-67 Breast Cancer Res Treat 2000 59 2 113 123 10.1023/A:1006344010050 10817346

[B50] Viale G Regan MM Mastropasqua MG Maffini F Maiorano E Colleoni M Price KN Golouh R Perin T Brown RW Kovács A Pillay K Ohlschlegel C Gusterson BA Castiglione-Gertsch M Gelber RD Goldhirsch A Coates AS International Breast Cancer Study Group Predictive value of tumor Ki-67 expression in two randomized trials of adjuvant chemoendocrine therapy for node negative breast cancer J Natl Cancer Inst 2008 100 3 207 212 10.1093/jnci/djm289 18230798

[B51] Bull SB Ozcelik H Pinnaduwage D Blackstein ME Sutherland DAJ Pritchard KI Tzontcheva AT Sidlofsky S Hanna WM Qizilbash AH Tweeddale ME Fine S McCready DR Andrulis IL The Combination of *p53 *Mutation and *neu/erb*B-2 Amplification Is Associated With Poor Survival in Node-Negative Breast Cancer Journal of Clinical Oncology 2004 22 1 86 96 10.1200/JCO.2004.09.128 14701769

[B52] Barbiti A Cosmi EV Sidoni A Collin P Parpora MG Ferri I Luthy M Lauro V Bucciarelli E Value of c-erbB-2 and p53 oncoprotein co-overexpression in human breast cancer Anticancer Res 1997 17 1A 401 405 9066684

[B53] Tan PH Bay BH Yip G Selvarajan S Tan P Wu J Lee CH Li KB Immunohistochemical detection of Ki67 in breast cancer correlates with transcriptional regulation of genes related to apoptosis and cell death Mod Pathol 2005 18 3 374 381 10.1038/modpathol.3800254 15578079

[B54] Kuroda H Ishida F Nakai M Ohnisi K Itoyama S Basal cytokeratin expression in relation to biological factors in breast cancer Hum Pathol 2008 39 12 1744 1750 10.1016/j.humpath.2008.06.007 18755493

[B55] Siziopikou KP Cobleigh M The basal subtype of breast carcinomas may represent the group of breast tumors that could benefit from EGFR-targeted therapies Breast 2007 16 1 104 107 10.1016/j.breast.2006.09.003 17097880

[B56] Atchley DP Albarracin CT Lopez A Valero V Amos CI Gonzalez-Angulo AM Hortobagyi GN Arun BK Clinical and pathologic characteristics of patients with BRCA-positive and BRCA-negative breast cancer J Clin Oncol 2008 26 26 4282 4288 10.1200/JCO.2008.16.6231 18779615 PMC6366335

[B57] Chen JQ Russo J ERalpha-negative and triple negative breast cancer: molecular features and potential therapeutic approaches Biochim Biophys Acta 2009 1796 2 162 175 19527773 10.1016/j.bbcan.2009.06.003 PMC2937358

[B58] Korsching E Jeffrey SS Meinerz W Decker T Boecker W Buerger H Basal carcinoma of the breast revisited: an old entity with new interpretations J Clin Pathol 2008 61 553 60 10.1136/jcp.2008.055475 18326009

